# Clinical and angiographic success and safety comparison of coronary intravascular lithotripsy: An updated meta-analysis

**DOI:** 10.1016/j.ijcha.2022.100975

**Published:** 2022-02-24

**Authors:** Yasar Sattar, Talal Almas, Junaid Arshad, Mohamed Zghouzi, Waqas Ullah, Tanveer Mir, Mohamed O. Mohamed, Islam Y. Elgendy, Wael Aljaroudi, Anand Prasad, Richard Shlofmitz, Mamas A. Mamas, Dean J. Kereiakes, M. Chadi Alraies

**Affiliations:** a Icahn School of Medicine at Mount Sinai Elmhurst Hospital, Queens, NY, USA; b Royal College of Surgeons in Ireland, Dublin, Ireland; c Pakistan Institute of Medical Science, Islamabad, Pakistan; d Detroit Medical Center, DMC Heart Hospital, Detroit, MI, USA; e Abington Jefferson Health, Abington, PA, USA; f Keele Cardiovascular Research Group, Keele University, Stoke-on-Trent, United Kingdom; g Weill Cornell Medicine-Qatar, Doha, Qatar; h Augusta University, Augusta, GA, USA; i UT Health San Antonio, San Antonio, TX, USA; j St Francis Hospital, The Heart Center, Roslyn, NY, USA; k The Christ Hospital Heart and Vascular Center, Cincinnati, OH, USA

**Keywords:** IVL, Intravascular lithotripsy, Coronary artery calcification, MACE, Major Adverse Cardiovascular Events, MI, Myocardial Infarction, TVR, Target Vessel Revascularization, CM, Cardiac Mortality, C-Dissection, Coronary Dissections, ST, Stent Thrombosis

## Abstract

**Background:**

Intravascular lithotripsy (IVL) can be used to assist stent deployment in severe coronary artery calcifications (CAC).

**Methods:**

Studies employing IVL for CAC lesions were included. The primary outcomes included clinical and angiographic success. The secondary outcomes, including lumen gain, maximum calcium thickness, and calcium angle at the final angiography site, minimal lumen area site, and minimal stent area site, were analyzed by the random-effects model to calculate the pooled standardized mean difference. Tertiary outcomes included safety event ratios.

**Results:**

Seven studies (760 patients) were included. The primary outcomes: pooled clinical and angiographic success event ratio parentage of IVL was 94.4% and 94.8%, respectively. On a random effect model for standard inverse variance for secondary outcomes showed: minimal lumen diameter increase with IVL was 4.68 mm (p-value < 0.0001, 95% CI 1.69–5.32); diameter decrease in the stenotic area after IVL session was −5.23 mm (95 CI –22.6–12.8). At the minimal lumen area (MLA) and final minimal stent area (MSA) sites, mean lumen area gain was 1.42 mm^2^ (95% CI 1.06–1.63; p < 0.00001) and 1.34 mm^2^ (95% CI 0.71–1.43; p < 0.00001), respectively. IVL reduced calcium thickness at the MLA site (SMD −0.22; 95% CI −0.40–0.04; P = 0.02); calcium angle was not affected at the MLA site. The tertiary outcomes: most common complication was major adverse cardiovascular events (n = 48/669), and least common complication was abrupt closure of the vessel (n = 1/669).

**Conclusions:**

Evidence suggests that IVL safely and effectively facilitates stent deployment with high angiographic and clinical success rates in treating severely calcified coronary lesions.

## 1 Introduction

Clinical Perspective

What is known:

• The most common cause of PCI failure can be secondary to severe coronary artery calcification.
• Athero-ablative procedures can be used to modify calcium, allowing more optimal stent delivery and expansion in such complex coronary cases. These adjuvant techniques are associated with an increased risk of complications while at the cost of stent under-expansion, stent damage, or malposition if the calcium modification is not optimal.

What is New

• Intravascular lithotripsy delivers circumferential, unfocused, and pulsatile energy to disrupt calcium within the target lesion safely with > 90% clinical and angiographic success. The purpose is to fracture the calcified plaque to allow proper expansion with a balloon to gain enough lumen diameter to pass and deploy the DES successfully.

What Are the Clinical Implications?

• Intravascular lithotripsy can offer a significant improvement in the vessel lumen to facilitate coronary stent delivery and deployments in severely calcified coronary arteries.

Percutaneous coronary intervention (PCI) failure is a clinical challenge of the coronary artery cases that require intervention. The most common cause of PCI failure can be secondary to severe coronary artery calcification [1], [2]. Athero-ablative procedures such as excimer lasers, rotational and orbital atherectomy, cutting balloons, and high-pressure non-compliant balloon catheters can be used to modify calcium, allowing more optimal stent delivery and expansion in such complex coronary cases [3]. These adjuvant techniques are associated with an increased risk of complications while at the cost of stent under-expansion, stent damage, or malposition if the calcium modification is not optimal. These complications secondary to PCI with atherectomy or assisted measures may be associated with increased rates of stent thrombosis and restenosis that may contribute to mortality [4], [5], [6].

Coronary intravascular lithotripsy (IVL) has been recently developed in order to manage coronary artery calcification. IVL delivers circumferential, unfocused, and pulsatile energy to disrupt calcium within the target lesion safely. The purpose is to fracture the calcified plaque to allow proper expansion with a balloon to gain enough lumen diameter to pass and deploy the DES successfully [7]. The ultrasonic waves travel through a balloon-based small size catheter disrupting only the superficial and deep calcium deposits with limited risk of vascular rupture or dissection [8], [9].

Intravascular lithotripsy has been shown to offer better safety and lower procedure-related complications. We sought to quantify an effect size through a *meta*-analysis of all available studies for IVL outcomes in severely calcified coronary lesions.

## 2 Methods

### 2.1 Data source

A bibliographical search of PubMed, EMBASE, Cochrane Central, and ClinicalTrials.gov databases for randomized clinical trials and observational studies was performed from inception until September 2020. The search items included medical subject headings (MeSH) and keywords: “intravascular shockwave lithotripsy,” “coronary lithotripsy,” “IVL,” “S-IVL,” “acute coronary syndrome,” “ST-elevation myocardial infarction,” “non-ST elevation myocardial infarction,” “unstable angina,” “stable angina,” “calcified coronary artery disease,” “failed percutaneous coronary intervention,” “stent under expansion,” and “drug-eluting stent,” These terms were combined using Boolean operators (“AND” or “OR”), and final results from all the possible combinations were downloaded into an EndNote library. Additional studies were identified by reviewing the reference lists of potentially relevant studies (**Supplementary S1**). Our study involved analysis of de-identified data and was exempt from institutional board review (IRB) approval.

### 2.2 Selection criteria

Studies were included if they met the following inclusion criteria: 1) studies that primarily evaluated coronary shockwave IVL, 2) studies of patients with coronary circulation calcified disease. For inclusion, the studies had to report data that evaluated the effectiveness of shockwave IVL for coronary circulation with baseline pre- and post-procedural changes in vessel diameter. Studies that included adjunctive modalities such as atherectomy and cutting balloons, but which primarily employed IVL with a goal to obtain gain in lumen features and stent optimization, were also included in the present analysis. Furthermore, studies that included a head-to-head comparison of atherectomy and cutting balloons were excluded from our quantitative analysis. We also excluded patients under 18 years of age from the present analysis.

### 2.3 Data extraction

Two authors (Y.S and J.A) independently reviewed the search results for studies that met the eligibility criteria. Uncertainty regarding study inclusion was resolved by consensus with a third author (W.U). The first phase of screening involved screening of titles and abstracts meeting selection criteria. The second screening phase required full-text reading of articles that identified items for data extraction based on the selection criteria. The mean and standard deviation of vessel characteristics pre-IVL, post-IVL, and post stenting were tabulated.

### 2.4 Outcomes

The primary outcomes for this analysis were clinical and angiographic success event ratios. The secondary outcomes included minimal lumen diameter (MLD), diameter stenosis (DS), lumen area, maximum calcium thickness, and calcium angle at minimal lumen area (MLA) and final minimal stent area (MSA). We also reviewed descriptive measures of final angiographic outcomes, including acute luminal gain, stent delivery, angiographic success, and clinical success. The safety measures studied were MACE (composite of myocardial infarction, target vessel revascularization, and cardiovascular mortality), coronary dissection, stent thrombosis, slow reflow, and no-reflow. *Clinical/Procedural success* was defined as ability to produce residual diameter stenosis < 50% in coronary artery by preventing major adverse cardiovascular events. *Angiographic success* was defined as successful stent delivery with a residual diameter stenosis value of 50% in the absence of significant procedural complications. Severe complications in all the studies included were defined as either angiographically (radio-opacities involving both sides of the arterial wall and length of at least 15 mm) or IVL (IVUS) or OCT [presence of ≥ 270 degrees of calcium on at least 1 cross-section].

### 2.5 Statistical analysis

This *meta*-analysis followed the Preferred Reporting Items for Systematic reviews and Meta-Analyses guidelines [10]. Statistical analysis was performed using the Cochrane Review Manager (RevMan) version 5.3. Data from each study that met the inclusion criteria were extracted into a table. The table's data elements included the study's country, age of participants, sex of participants, sample size, comorbidities of participants, procedural parameters, safety, and follow-up. Data reported in median was converted to mean with standard deviation using a standardized Hozo equation [11]. Outcome variables were analyzed by a random-effects model (inverse variance) to calculate the pooled standardized mean difference with a statistical significance of probability value p < 0.05 [12]. The “test for overall effect” was reported as z value corroborating the 95% confidence interval's inference. Descriptive statistics were performed for outcome measures that did not have data in a format to perform mean inverse variance.

Higgins I-squared (I^2^) was determined as a measure of statistical heterogeneity where values of ≤ 50% corresponded to low to moderate heterogeneity while values ≥ 75% indicated high heterogeneity [13]. The publication bias was depicted graphically and numerically as a forest plot and Egger’s Regression Equation (ERE) [14]. The included articles' quality assessment was performed using the Cochrane guidelines for the systematic review and *meta*-analysis, and Newcastle–Ottawa scale (NOS), where each study was screened for five different types of bias (selection, performance, detection, attrition, and reporting bias). The NOS scale score ranged from 0 to 9; a score of 7 and above is considered high quality.

## 3 Results

**Fig. 1:**
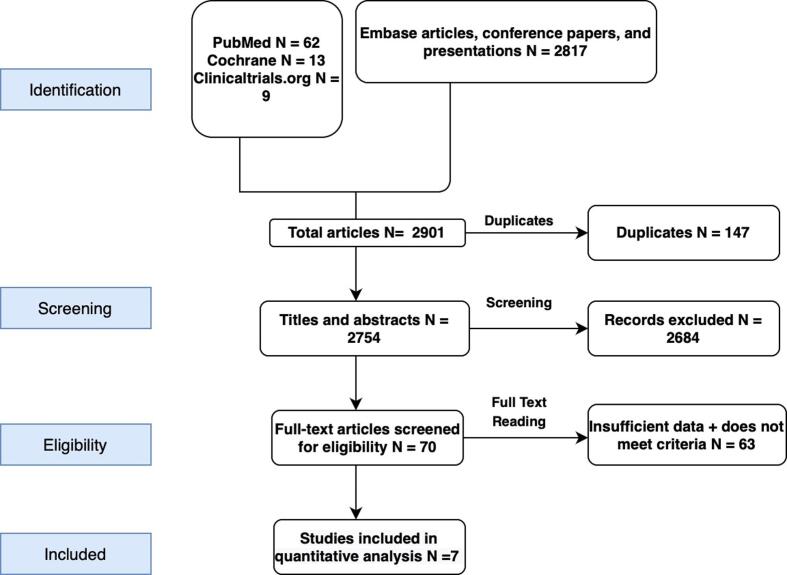
PRISMA Flow of the search strategy for systematic review and *meta*-analysis Caption. PRISMA flow diagram is a summary of systematic search methods that explain how the eligible studies were screened and included in analysis. N; number of articles.

**INCLUDED STUDIES:** The electronic search resulted in 2,901 articles that underwent title and abstract screening. Following duplicate (n = 149) removal and irrelevant items (n = 2687), 70 articles were reviewed in full-text form. Based on the selection criteria, 7 original studies, including one subgroup study, qualified for final analysis [15], [16], [17], [18], [19], [20], [21]. The detailed search strategy is shown in the PRISMA flow diagram (Fig. 1). One study included in-stent coronary artery calcification and used IVL in this population [19]. All the studies other than SMILE registry used IVL on coronary artery calcification and then underwent stent deployment [15], [16], [17], [18], [20]. A total of 760 patients were included in our study. The mean age of the population was 72.4 years. The most common comorbidity was hypertension. The most common target vessel was the left anterior descending artery. Severe and moderate calcifications were present in up to 94% and 6% of cases, respectively. The mean balloon size, pressure, and catheter size was 4.0 × 12 mm, 6 atm, and 6fr, respectively. The baseline demographic, lesion, and procedural characteristics are shown in Table 1. The included studies' methodological quality was moderate based on the mean Newcastle Ottawa Scale (NOS) score of 6. The detailed NOS is given in **Supplementary S2.**

**Table 1:** Baseline demographics and comorbidities of the study population.

**Author**	**Brinton (DISRUPT CAD I) 2017 ^15^**	**Ali ZA (DISRUPT CAD OCT sub-group) – 2017 ^16^**	**Ali (DISRUPT CAD II) – 2019 ^17^**	**Aksoy 2019 ^18^**	**Hill (DISUPT CAD III) 2020 ^20^**	**Lelasi (SMILE) 2020 ^19^**	**DISRUPT CAD IV ^21^**
Country	Multi-national	Multi-national	Multi-national	Germany	US, UK, France & Germany	Italy	Japan
Age (Years)	73 ± 7	71 ± 10	72 ± 10	76 ± 10	71.2 ± 8.6	69.2 ± 3.8	62.3 ± 4.5
Male % (n)	80 (48)	80 (25)	78 (94)	51 (72)	76.6 (294)	76 (26)	72 (63).
Sample Size (n)	60	31	120	71	384	34	
ACS % (n)	40 (24)	42 (13)	26 (31)	45 (32)	18.0(69)	44.1(15)	34.2 (32)
HTN % (n)	80 (48)	24 (77)	80 (96)	93 (66)	89.1(342)	85.2(29)	74.3 (21.3)
DM % (n)	30 (18)	7 (23)	32 (38)	34 (24)	40.1(154)	52.9(18)	23.1 (23)
Dyslipidemia % (n)	80 (48)	26 (83)	72 (86)	62 (45)	89.1(342)	82.3(28)	72 (49)
Smoking % (n)	15 (9)	7 (23)	13 (16)	37 (26)	12.2(47)	50(17)	
TIA/Stroke	13 (8)	–	3 (4)	17 (12)	7.6 (29)		
Angina Classification							
0					48/381 (12.6)		
I	19 (32)	13 (42)			56/381 (14.7)		
II	29 (48)	16 (52)			142/381 (37.3)		
III	10 (17)	1 (3)			126/381 (33.1)		
IV	2 (3)	1 (3)			9/381 (2.4)		

Abbreviations: ACS: Acute Coronary Syndrome; HTN: Hypertension; DM: Diabetes Mellitus; TIA: Transient Ischemic Attack.

### 3.1 Primary outcome

**Fig. 2:**
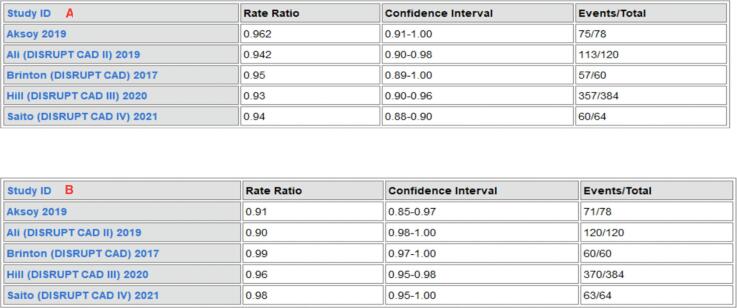
Event Ratios of Clinical/Angiographic and Procedural Success in IVL patients.

The event ratios of pooled clinical/procedural (success events n = 662/sample total706). and angiographic success (success events n = 684 /sample total n = 706) was 94.4% and 94.8%, respectively **(**Fig. 2**).**

### 3.2 Secondary outcome efficacy of pre- and post- intravascular lithotripsy in calcified coronary disease on angiography

**Fig. 3:**
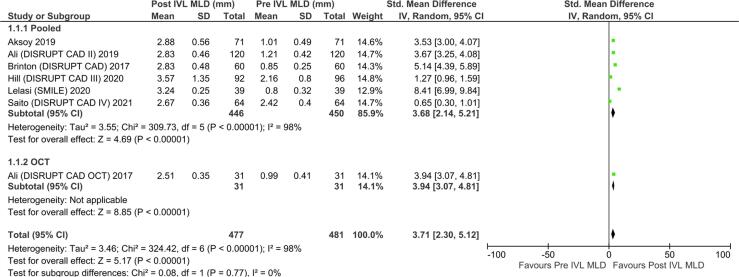
Forest plot showing outcomes of minimal lumen diameter (MLD) in patients that received IVL as compared to pre-IVL.

**Fig. 4:**
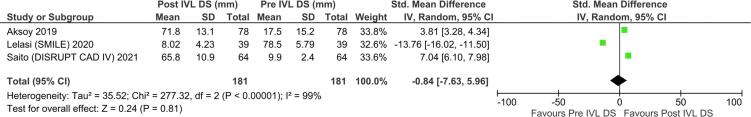
Forest plot showing outcomes of diameter stenosis in patients that received IVL as compared to pre-IVL.

A total of 483 and 467 patients were included in comparing MLD in pre-IVL and post IVL groups, respectively. The overall MLD diameter change with IVL was 4.68 mm (p-value < 0.0001, 95% CI 1.69–5.32) **(**Fig. 3**).** Sensitivity analysis was performed, and I2 dropped to 0% by removing study Brinton DISRUPT CAD I, Lelasi SMILE, Saito DISRUPT CAD 4, and Hill DISRUPT CAD III **(Supplementary S3)**. The likely cause of heterogeneity caused by these studies was due to selection bias given the variation in sample size and angiographic features. A total of 181 patients in each pre-IVL and post-IVL group were included for comparison of DS. The overall DS change in the stenotic area after the IVL session was −0.84 (95% CI: −7.63 to 5.96) **(**Fig. 4**)**. The mean acute luminal gain in post IVL and post-stent was 1.21 mm and 1.89 mm, respectively (see Table 2.).

**Table 2:** Angiographic lesion characteristics, and procedural characteristics of the study population.

**Author**	**Brinton (DISRUPT CAD I) 2017 ^15^**	**Ali ZA (DISRUPT CAD OCT sub-group) – 2017 ^16^**	**Ali (DISRUPT CAD II) – 2019 ^17^**	**Aksoy 2019 ^18^**	**Hill (DISUPT CAD III) 2020 ^20^**	**Lelasi (SMILE) 2020 ^19^**	**DISRUPT CAD 4 ^21^**
**Lesion Angiographic Characteristics**							
Calcium angle definition	Calcific angle was defined as low-attenuation signal with sharply delineated borders	Calcification angle was measured using a protractor centered on the lumen. If there were > 1 calcium deposits present in a single cross-sectional frame, the angle was defined as the sum of the angles of each individual calcium deposit for that cross section.	Calcification angle was measured using a protractor centered on the lumen. If there were > 1 calcium deposits present in a single cross-sectional frame, the angle was defined as the sum of the angles of each individual calcium deposit for that cross section.	–	Max calcium site was defined as the site with maximum calcium arc	–	Key parameter, such as calcium angle, were determined and defined using OCT
Protected LMA n (%)	1(2)		1 (0.8)	13 (16.7)	6 (1.6)		7 (4)
LAD	28 (47)	14 (45.1)	75 (62.5)	34 (43.6)	217 (56.5)	18 (46.1)	21 (54.3)
Circumflex	8 (13)	5 (16.1)	14 (11.7)		49(12.8)	3(7.6)	8 (8.3)
RCA	23 (38)	12 (38.7)	30(25)	26 (33.3)	112(29.2)	5(12.8)	3 (3.4)
Lesion Localization							
Ostial				18 (23.1)	0/111 (0.0)		
Proximal				31 (39.7)	31/111 (27.9)		
Mid				26 (33.3)	53/111 (47.7)		
Distal				3 (3.8)	27/111 (24.3)		
Reference vessel diameter, mm (median IQR range, or mean ± SD)	3 (2.6–3.2)	2.87 ± 0.49	3.04 ± 0.53		3.03 ± 0.47	3.27 ± 0.25	
Minimal lumen diameter, mm (median IQR range, or mean ± SD)	0.9 (0.6–1.1)	0.99 ± 0.41	1.21 ± 0.42		1.06 ± 0.36 [381]	0.80 ± 0.3	
Diameter stenosis % (median IQR range, or mean ± SD)	73 (59–77)	65.1 ± 14.4	60.0 ± 12.0		65.1 ± 10.8 [381]	78.5 ± 5.8	78.5 ± 5.8
Lesion length, mm (mean ± SD)	18 (14–25)	21.7 ± 11.6	19.5 ± 9.8	21 ± 16	26.0 ± 11.7 [381]		
Calcified length, mm (mean ± SD)	21 (12, 25)	21.3 ± 10.3	25.7 ± 12.4		47.9 ± 18.8		
Calcifications: severe (s) n (%), moderate (m) (n (%)	S 60 (1 0 0)	S 27 (87.1)	S 113 (94.2)	S 64 (82.1), m 14 (17.9)	384 (100.0)	33(84.6)	
Concentric calcification n (%)	47 (78)		86 (71.7)				46 (74.4)
Eccentric calcification n (%)	13 (22)		34 (28.3)				
Bifurcation lesion with side branch involvement (bi) n (%), CTO n (%)	Bi 17 (28)		Bi 36 (30)	CTO 2 (2.7)	Bi 115 (29.9)		
**Procedural characteristics**							
	
Total procedure time min	92 (70–109)	92.9 ± 36.0	68.3 ± 34.2		53.0 (38.0–74.0)		
Fluoroscopy time min	27 (18–41)	31.0 ± 15.0	18.0 ± 11.1	27.34 ± 18.95	15.0 (11.0–24.0)	26.5 ± 6	
Contrast volume, ml		294.0 ± 95.9	181.9 ± 66.4	165 ± 63	167.9 ± 71.9	167.3 ± 32	
Device time, min			7.9 ± 5.2				
No. of lithotripsy catheters (median with IQR range or mean ± SD)	2 (1, 2)	2(1–2)	1.2 ± 0.6		1.2 ± 0.5		
IVL Pressure pre/post IVL, atm	6/6, atm	4/6, atm	4/6, atm	4/6, atm	6/6, atm		
Number of IVL Pulses	72 (40–120)	94.0 ± 75.0	70.7 ± 43.4	68 ± 25	68.8 ± 31.9	55.8 ± 14.4	
Pre-dilation n (%)	22 (37)		50 (41.7)	32 (41.0)	212 (55.2)	39(100)	
Post IVL Dilation n (%)	52 (87)		95 (79.2)	25 (32.1)	78/377 (20.7)	29(74.3)	
Catheter Size (French Fr)	6F	6F	6F	6F (18% 7F)	7F		
Access TR (n) TF (n) TU (n) TB (n)	TR or TF	TR or TF	TR or TF	TF (47), TR (29.5)	TF (154), TR (227) TU (1), TB (2)		
Max IVL Inflation pressure atm			5.8 ± 0.7		6.0 ± 0.3	6.0 ± 1.2	
OCT Guided		6 (19)		12 (15.4)		9 (23.1)	
No of Stents implanted per lesion (mean)	1 (1, 2)	1 (1–2)	1.3 ± 0.6	1.3			
0					3 (0.8)		
1					289 (75.3)		
2					85 (22.1)		
3					7 (1.8)		
Post stent dilation balloons used n (%)		28 (90)		37 (47.4)	377/381 (99.0)		
Largest diameter of post stent dilation balloon (mean with SD) mm				3.8 ± 1.0			
Post stent dilation mean pressure, atm		30.7 ± 11.9		17 ± 5			
Total stent length, mm		31.0 ± 12.0			31.0 ± 12.0	26.7 ± 4	
Duration of hospitalization, days		1 (1–1)			2.0 (1.0, 2.0)		

Abbreviations: LMA: Left Main Artery; LAD: Left Anterior Descending; RCA: Right Coronary Artery; IVL: Intravascular Lithotripsy; TR: Trans-Radial; TF: Trans-Femoral; TU: Trans-Ulnar; TB: Trans-Brachial; OCT: Optical Coherence Tomography.

### 3.3 Pre- and post- intravascular lithotripsy efficacy at minimal lumen area

**Fig. 5:**
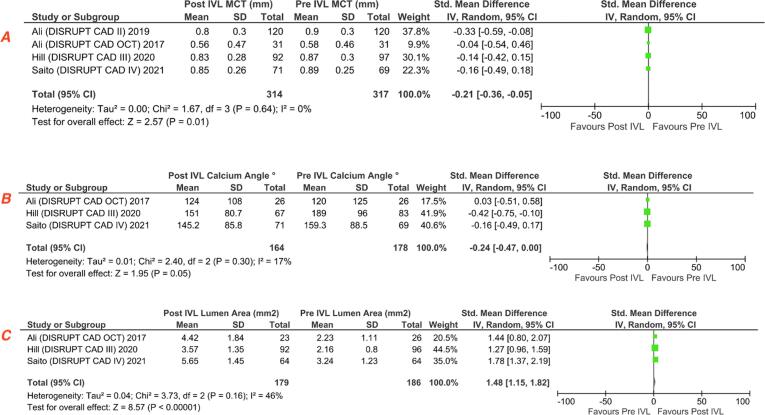
Forest plot showing standard mean differences of lumen area at minimal lumen area (MLA) **(A)**, maximum calcium thickness (MCT) **(B)**, maximum calcium angle **(C)** among pre-IVL and post-IVL.

The MLA in pre-IVL (n = 122) and post-IVL(n = 115) showed a mean gain of 1.31 mm^2^ (95% CI 1.02–1.59; P < 0.00001) **(**Fig. 5**A)**. Furthermore, statistically significant difference in the maximum calcium thickness (SMD −0.22; 95% CI −0.40–0.04; P = 0.02) while calcium angle among pre- and post-IVL at MLA site was not statistically significant (SMD −0.25; 95% CI −0.68–0.19; p = 0.26) **(**Fig. 5**B-C)**.

### 3.4 Pre- and post- intravascular lithotripsy efficacy at final minimal stent area

**Fig. 6:**
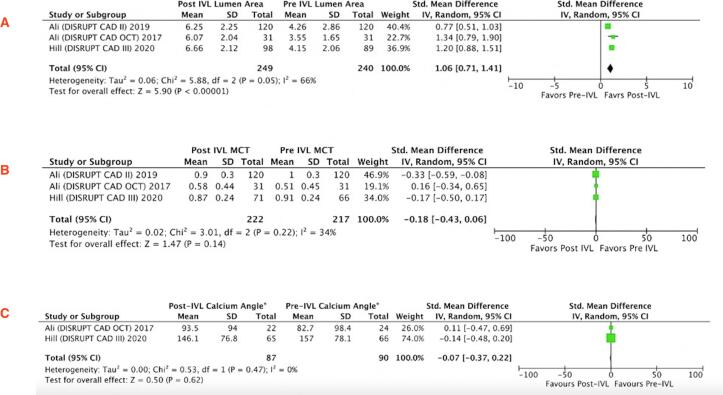
Forest plot showing standard mean differences of lumen area at minimal stent area (MSA) **(A)** ,maximum calcium thickness (MCT) **(B)**, maximum calcium angle **(C)** among pre-IVL and post-IVL.

The mean lumen area for the MSA site in pre-IVL (n = 240) and post-IVL(n = 249) showed a mean gain of 1.06 mm^2^ (95% CI 0.71–1.41; P < 0.00001; I2 = 66%). There was no statistically significant difference of maximum calcium thickness (SMD −0.18; 95% CI −0.43–0.06; p = 0.14; I2 = 34%) and calcium angle (SMD −0.07; 95% CI −0.37–0.22; p = 0.62) among pre- and post-IVL at final MSA site **(**Fig. 6**A-C).**

### 3.5 Intravascular lithotripsy safety

**Fig. 7:**
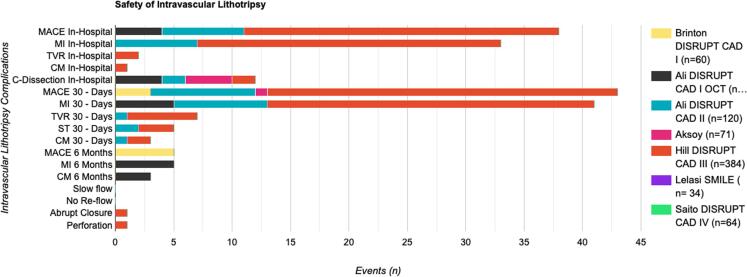
Stacked bar chart showing complications of intravascular lithotripsy with events shown as bar extension and percentages of events on top of stack bar.

In general, the complication rate was low. In 669 patients, the most common complication was MACE at 30-day follow-up (n = 48). During the 30-day follow-up, myocardial infarction, including periprocedural MI (n = 44) and target vessel revascularization (n = 7), were the two leading MACE constituents. In-hospital MACE (n = 39) were the second most complications. Among in-hospital MACE, periprocedural MI (n = 34) was the leading MACE component. Coronary dissection (n = 12) was the second most common in-hospital complication. The abrupt closure of vessels and perforation were the least common complications. None of the studies reported any slow reflow or no-reflow events after IVL sessions. A summary of IVL complications based on follow-up are shown in Fig. 7**.**

**PUBLICATION BIAS:** The plot's vertical axis used standard error to estimate the study's sample size, plotting large population studies on top and smaller at the bottom. The horizontal spread reflected the power and effect size of the included studies. Our funnel plot was not symmetrical on visual assessment, indicating that the limited scatter might be due to publication bias **(Supplementary S4)**. The numerical assessment of publication bias was done using Egger’s regression model that failed to show any publication bias or small study effects (ERE ≈ p > 0.05). Furthermore, the heterogeneity among the outcomes of the included studies was self-explicable. First, as per the Cochrane handbook of the systematic review and *meta*-analysis, if the total count of included studies is less than ten, it is not possible to differentiate between true heterogeneity and findings merely by chance. Second, while all the studies unanimously supported the IVL, the sampling error could explain the high percentage of variability.

## 4 Discussion

This comprehensive *meta*-analysis of 7 studies with 760 patients, IVL enhanced MLD and lumen area at MLA and final MSA site. The pooled clinical and angiographic success of IVL is 94%. However, IVL was not associated with a change in diameter stenosis, calcium angle at any vessel point except maximum calcium thickness at the MLA site. The most common complications in-hospital and to 30-days were including periprocedural MI and coronary dissection.

Coronary artery calcifications may pose a challenge to the successful delivery of stent platforms, and even if successfully delivered, may result in stent under-expansion, which may, in turn, might lead to further complications including MACE, restenosis, target lesion failure, and stent thrombosis. Coronary IVL is a contemporary intervention that uses ultrasonic waves without athero-ablation to target superficial and deep layers of calcium in coronary artery calcification. Owing to ease of use, the low-pressure balloon inflation, low frequency of serious complications, and small (6F) guide catheter requirement, IVL is preferred over prior calcium debulking techniques. Our *meta*-analysis findings suggest that IVL might be an effective adjunctive procedure to help with coronary artery calcifications.

Brinton et al. DISRUPT CAD I was the first study to assess the effectiveness of IVL for coronary artery calcification [15]. A total of 60 patients were enrolled from seven different hospitals in five countries. The included study population had ≥ 1 lesion requiring PCI with heavy calcification, diameter stenosis ≥ 50%, and lesion length ≤ 32 mm. The study's primary outcome was to assess clinical efficacy: the ability to reduce mean diameter stenosis < 50% with no evidence of MACE at 30 days follow-up. DISRUPT CAD results showed a mean decrease of calcium angle, calcium thickness, lumen area was 24°, 0.03 ± 0.01 mm, and 4.24 ± 2.34 mm, respectively. Calcium fracture was achieved in > 25% of the lesions with a mean acute diameter gain of 2.1 mm. The study resulted in favorable outcomes to deploy stents in up to 95% of patients who underwent IVL. Subsequently, Ali et al. performed a subgroup study of CAD I using optical coherence tomographic (OCT) imaging for reporting the angiographic outcomes of IVL [16]. The Authors included 31 patients in subgroup analysis that underwent IVL and resulted in a calcium fracture that was highest in heavily calcified lesions (highest vs. lowest percentile of calcium: 77.8 % vs. 22.2 %, p = 0.057). The mean acute luminal after IVL was 2.1 mm^2^, which can be expanded to 5.94 ± 1.98 mm^2^ with DES. The mean stent expansion was 112.0 ± 37.2%. Deep dissection, as part of the IVL procedure, was seen in 13% of the cases. The population analyzed did not experience vessel perforation, slow flow/no reflow, or closure after the IVL procedure. Furthermore, DISRUPT CAD II data on IVL was also reported by the same authors (ALI et al.), who assessed the efficacy of IVL in severely calcified lesions [17]. The study's primary outcome was MACE (target vessel revascularization, myocardial infarction, and cardiac mortality) after IVL. This study reported a mean drop in calcium angle, calcium thickness, lumen area by 51°, 0.04 ± 0.2 mm, and 4.83 ± 3.04 mm, respectively. The post-IVL acute luminal gain was 0.83 ± 0.47 mm that increased to 7.7 ± 7.1% by DES implantation with residual stenosis of 32.7 ± 10.4%. IVL associated MACE developed in 7 (5.8%) patients with non-Q wave myocardial infarction. The rest of the complication review assessment at 30 days follow-up showed no perforation, dissection, or early in-stent stenosis. The most recent study performed by Aksoy et al. in 2019 [18] reported an improvement in stenosis from a baseline of 71.8 ± 13.1% to 45.1 ± 17.4% after IVL and 17.5 ± 15.2% after stenting. Luminal diameter improvement from 1.01 ± 0.49 mm to 1.90 ± 0.61 with IVL and 2.88 ± 0.56 mm after stenting. An 84.6% success rate has been reported in patients who had IVL as their primary procedure. The Intravascular lithotripsy for the Management of un-dilatable coronary stent: (SMILE) study assessed the safety of IVL in calcified in-stent restenosis. The study reported that IVL was a success in 87.1% of cases with significant improvement in minimal stent diameter (pre IVL 0.81 mm; post IVL 3.23 mm; p = 0.00001) and minimal stent cross-sectional area (pre IVL 3.35 mm; post-IVL 7.61 mm; p = 0.00001) [19]. In September 2020 CAD III prospective study by Hill et al. 431 patients were enrolled from 47 sites in the US, UK, France, and Germany. CAD III was a non-randomized, single-arm study with performance safety and efficacy goals established from the ORBIT II pivotal trial for orbital atherectomy approval. The primary safety measure was freedom from MACE to 30 days after the procedure. The primary efficacy outcome of the study was a procedural success without in-hospital MACE. An OCT sub-study in 100 patients was also reported [20]. The study reported that 92.2% of the study population were free from MACE within 30 days of IVL (p < 0.0001 95% CI), and procedural success was achieved in up to 92.4% of patients. The mean calcium length, calcium thickness, and calcium angle was 47.9 ± 18.8 mm, 0.96 ± 0.25 mm, and 292.5 ± 76.5° respectively. Our results are consistent with previous studies reporting an increase in MLD, lumen area at MLA site, and MSA site. Furthermore, IVL facilitates low-pressure IVL balloon inflation (6 atm), which achieves a remarkable increase in MLD and restenosis reduction. This is achieved because of calcium fracture and mitigation of fibro-elastic recoil. Fracture expansion occurs with subsequent stent implant [8].

Multiple studies have reported different trends of complications of IVL [15], [16], [17], [18], [19], [20], [21]. In terms of complications, MACE reported in most studies is a combination of cardiac death, TVR, MI, and coronary artery dissection. The complications of IVL are most commonly due to balloon inflation or balloon rupture. We found that MI was the most common complication after IVL at the in-hospital, 30 days, and six-month follow-up. Coronary artery dissection type B is the second most common complication after IVL.

The major limitation of all studies was the small sample size and non-randomized control groups. Our *meta*-analysis pooled all studies to increase the sample size to refine summary estimates to better assess the safety and effectiveness of IVL. 30-day and 6-month outcomes from available data show the capacity of IVL to successfully improve the expansion of the vessel to deploy stents with minimal vessel wall injury and lower MACE. Our study also highlights the paucity of clinical studies and the need for further controlled studies on longer follow-up duration.

DISRUPT CAD IV (NCT04151628): was a prospective single-arm study that enrolled 72 patients from Japan. Subjects were followed at discharge, 30 days, 6,12, and 24 months. Results from the CAD IV trial revealed a notable calcium length of 49.8 ± 15.5 mm. Additionally a calcium angle of 257.9 ± 78.4° was concluded by the study, exhibiting high rates of procedural success and low rates of major adverse cardiac events [21]. Another ongoing study, RAINBOW (NCT04013906) trial, a randomized clinical trial to evaluate the plaque modification after rotational atherectomy vs. IVL before DES implantation.

### 4.1 Limitations

Due to limited data, only single-arm observational studies were included; more studies, including randomized, double-blind studies, should be performed to study the safety and efficacy in a head-to-head comparison with other calcium debulking procedures. Severe calcification definition was not uniform in included studies given lack of consistency of imaging use including intravascular ultrasounds and optical coherence tomography. Our result of diameter stenosis had high heterogeneity, which cannot be excluded given only 2 studies reported data. Additionally, none of the included studies afforded adjunctive treatment with atherectomy or specialty cutting balloons. The post-procedural outcomes obtained therefore did account for any form of adjunctive treatment. Our study predominantly discussed the angiographic comparison of lesion outcomes pre- and post- IVL. As such, the studies included did not allow adjunctive treatment with atherectomy or specialty cutting balloons. Currently, there is no RCT head-to-head comparison of atherectomy (orbital or rotational) or cutting balloons with IVL.

## 5 Conclusion

Intravascular lithotripsy can offer a significant clinical and angiographic success with improvement in the vessel lumen to facilitate coronary stent delivery and deployments in severely calcified coronary arteries.

## Funding

This study was not financially supported. No funding organization or sponsor was involved in the design and conduct of the study; collection, management, analysis, and interpretation of the data; preparation, review, or approval of the article; and decision to submit the article for publication.

## Declaration of Competing Interest

The authors declare that they have no known competing financial interests or personal relationships that could have appeared to influence the work reported in this paper.
