# Supplementary material for: Clinical and angiographic success and safety comparison of coronary intravascular lithotripsy: An updated meta-analysis
Source: Int J Cardiol Heart Vasc. 2022 Feb 24;39:100975. doi: 10.1016/j.ijcha.2022.100975 (PMC8881660; doi:10.1016/j.ijcha.2022.100975)

**Supplementary file**

**Supplementary S1: Search Strategy**

**MeSH +Keyword:**

Intravascular shockwave lithotripsy
Shockwave intravascular lithotripsy

Coronary lithotripsy

IVL

S-IVL

Acute coronary syndrome

ST-elevation myocardial infarction

Non-ST elevation myocardial infarction

Unstable angina

Stable angina
Calcified coronary artery disease

Failed percutaneous coronary intervention

Stent under expansion

Drug eluting stent

Failed rotational atherectomy

**PubMed**

(((((Intravascular shockwave lithotripsy) OR (Shockwave intravascular lithotripsy)) OR (coronary lithotripsy)) OR (IVL)) OR (S-IVL)) AND ((((((((((Acute coronary syndrome) OR (ST-elevation myocardial infarction)) OR (Non-ST elevation myocardial infarction)) OR (Unstable angina)) OR (Stable angina)) OR (Calcified coronary artery disease)) OR (Failed percutaneous coronary intervention)) OR (Stent under expansion)) OR (Drug eluting stent)) OR (Failed rotational atherectomy))

**Embase Classic+ Embase**

| **#** | **Searches** | **Results** |
| --- | --- | --- |
| 1 | exp lithotripsy/ | 14948 |
| 2 | coronary lithotripsy.kw. | 3 |
| 3 | Intravascular shockwave lithotripsy.tw. | 2 |
| 4 | Intravascular lithotripsy.kw. | 28 |
| 5 | Shockwave lithotripsy.kw. | 428 |
| 6 | S-IVL.mp. | 49 |
| 7 | IVL.tw. | 927 |
| 8 | IVL.kw. | 21 |
| 9 | exp acute coronary syndrome/ or exp coronary artery disease/ or exp ischemic heart disease/ or exp non st segment elevation acute coronary syndrome/ | 854559 |
| 10 | calcified coronary artery disease.mp. or coronary artery calcification/ | 5440 |
| 11 | exp st segment elevation myocardial infarction/ or exp heart infarction/ | 409023 |
| 12 | exp non ST segment elevation myocardial infarction/ | 15192 |
| 13 | exp unstable angina pectoris/ | 24390 |
| 14 | exp stable angina pectoris/ | 11539 |
| 15 | Failed percutaneous coronary intervention.kw. | 0 |
| 16 | exp percutaneous coronary intervention/ or exp interventional cardiovascular procedure/ or exp transluminal coronary angioplasty/ | 148096 |
| 17 | exp drug eluting stent/ or exp stent/ or exp sustained release preparation/ or exp drug eluting cardiovascular stent/ or exp drug eluting digestive stent/ or exp drug eluting nitinol stent/ or exp drug eluting sinus stent/ or exp drug eluting tracheobronchial stent/ or exp drug eluting ureter stent/ or exp drug eluting urethral stent/ | 203824 |
| 18 | exp atherectomy/ or exp artery surgery/ or exp interventional cardiovascular procedure/ or exp coronary atherectomy/ or exp directional atherectomy/ or exp rotational atherectomy/ | 309653 |
| 19 | 1 or 2 or 3 or 4 or 5 or 6 or 7 or 8 | 16086 |
| 20 | 9 or 10 or 11 or 12 or 13 or 14 or 15 or 16 or 17 or 18 | 1130411 |
| 21 | 19 and 20 | 2817 |

**Cochrane Central Database: Date Run: 10/10/2020 00:26:53**

| **#** | **Searches** | **Results** |
| --- | --- | --- |
| 1 | Intravascular shockwave lithotripsy | 6 |
| 2 | Shockwave intravascular lithotripsy | 6 |
| 3 | Coronary lithotripsy | 14 |
| 4 | IVL | 39 |
| 5 | S IVL | 14 |
| 6 | Intravasc* Lithotri* | 11 |
| 7 | Acute coronary syndrome | 6791 |
| 8 | ST-elevation myocardial infarction | 3121 |
| 9 | Non-ST elevation myocardial infarction | 1616 |
| 10 | Unstable angina | 4286 |
| 11 | Calcified coronary artery disease | 198 |
| 12 | percutaneous coronary intervention | 10,644 |
| 13 | Stent under expansion | 48 |
| 14 | Drug eluting stent | 3941 |
| 15 | atherectomy | 462 |
| 16 | #1 OR #2 OR #3 OR #4 OR #5 OR #6 | 53 |
| 17 | #7 OR #8 OR #9 OR #10 OR #11 OR #12 OR #13 OR #14 OR #15 | 21449 |
| 18 | #16 AND #17 | 13 |

ClinicalTrials.gov

| **Terms** | **Search Results*** | **Entire Database**** |
| --- | --- | --- |
| Synonyms | | |
| **lithotripsy** | 9 studies | 180 studies |
| Litholapaxies | -- | 1 studies |
| **calcified coronary artery disease** | -- | 1 studies |
| **coronary artery disease** | 9 studies | 7,797 studies |
| Coronary Disease | 9 studies | 4,689 studies |
| myocardial ischaemia | 9 studies | 7,488 studies |
| Atherosclerosis of coronary artery | -- | 4 studies |
| Atherosclerosis of native coronary artery | -- | 1 studies |
| Atherosclerotic heart disease | -- | 16 studies |
| cardiac ischaemia | -- | 28 studies |
| Coronary Arterioscleroses | -- | 149 studies |
| coronary artery atherosclerosis | -- | 3 studies |
| coronary atheroma | -- | 7 studies |
| Coronary Atheroscleroses | -- | 136 studies |
| coronary heart disease | -- | 705 studies |
| Disease coronary artery | -- | 1 studies |
| ischemic heart disease | -- | 433 studies |
| **artery disease** | 9 studies | 6,245 studies |
| arterial diseases | -- | 1,640 studies |
| arterial vascular disease | -- | 3 studies |
| Arteriopathic disease | -- | 1 studies |
| Arteriopathy | -- | 19 studies |
| artery disorders | -- | 1 studies |
| **coronary artery** | 9 studies | 5,037 studies |
| **disease** | 9 studies | 257,226 studies |
| condition | -- | 11,993 studies |
| Disorders | -- | 92,462 studies |
| **artery** | 9 studies | 11,114 studies |
| Arterial | 8 studies | 9,016 studies |
| Arteria | -- | 7 studies |
| **coronary** | 9 studies | 24,863 studies |
| Heart | 9 studies | 21,313 studies |
| Cardiac | -- | 9,787 studies |
| **calcified** | 9 studies | 325 studies |
| Calcification | 2 studies | 235 studies |
| calcific | -- | 58 studies |
| calcify | -- | 1 studies |
| calcifying | -- | 4 studies |

| -- | No studies found |
| --- | --- |
| * | Number of studies in the search results containing the term or synonym |
| ** | Number of studies in the entire database containing the term or synonym |

**Supplementary S2: Table 2**

| **Table showing Newcastle-Ottawa Scale (NOS) for assessing the quality of nonrandomized studies in meta-analysis** | | | | | | | | | |
| --- | --- | --- | --- | --- | --- | --- | --- | --- | --- |
| **Selection** | | | | | | **Outcome** | | | |
| Study | Representativeness of the exposed cohort | Selection of the non-exposed cohort | Ascertainment of exposure | Outcome not present at baseline | Comparability  of the  cohort | Assessment  of  outcome | Enough  follow-  up  duration | Adequate  follow-  up | Total  score |
| Brinton  (DISRUPT  CAD) 2017 | * | N/A | * | * | N/A | * | * | * | 6 |
| Ali  (Disrupt CAD  OCT) 2017 ^10^ | * | * | * | * | N/A | * | * | * | 6 |
| Ali  (Disrupt CAD-  II) 2019 ^11^ | * | * | * | * | N/A | * | * | * | 6 |
| Aksoy  2019 ^12^ | * | N/A | * | * | N/A | * | * | * | 6 |
| Lelasi SMILE 2020 | * | * | * | * | N/A | * | * | * | 6 |
| Hill (DISRUPT CAD III) 2020 | * | * | * | * | N/A | * | * | * | 6 |
|  |  |  |  |  |  |  |  |  |  |
| DISRUPT CAD 4 |  |  |  |  |  |  |  |  |  |
|  |  |  |  |  |  |  |  |  |  |
|  |  |  |  |  |  |  |  |  |  |

**Supplementary S3: Sensitivity Analysis**

**
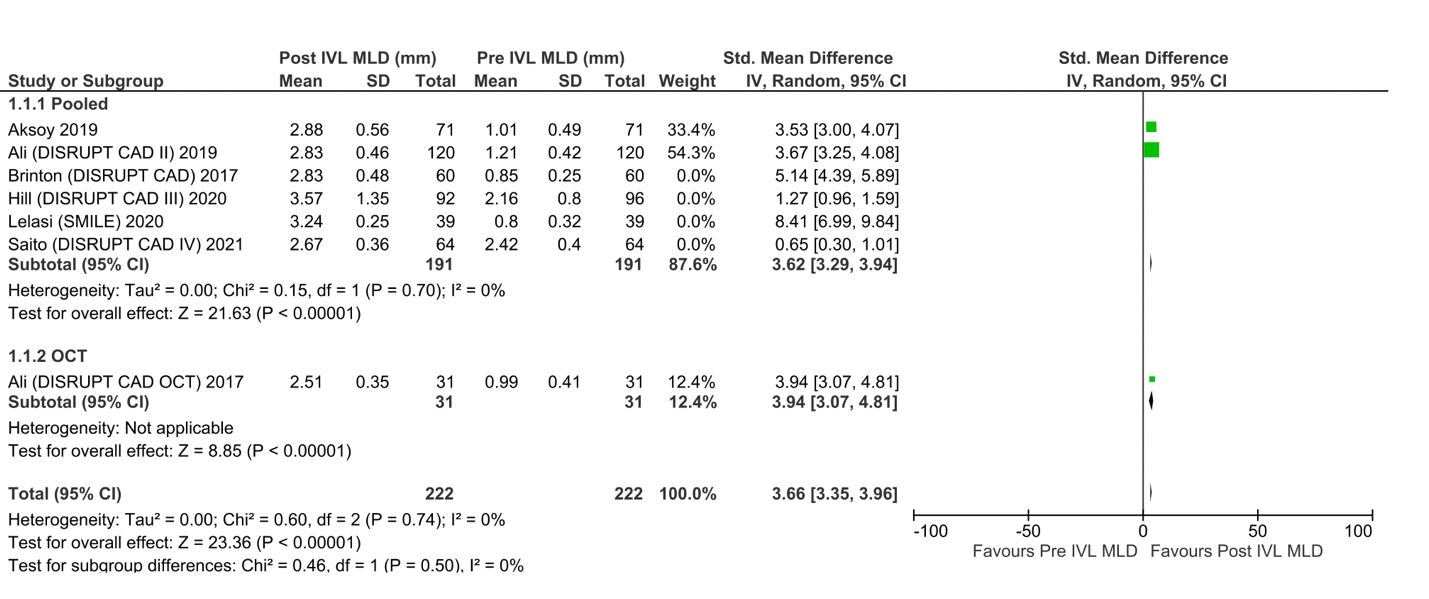
**

**Supplementary S4: Publication bias**


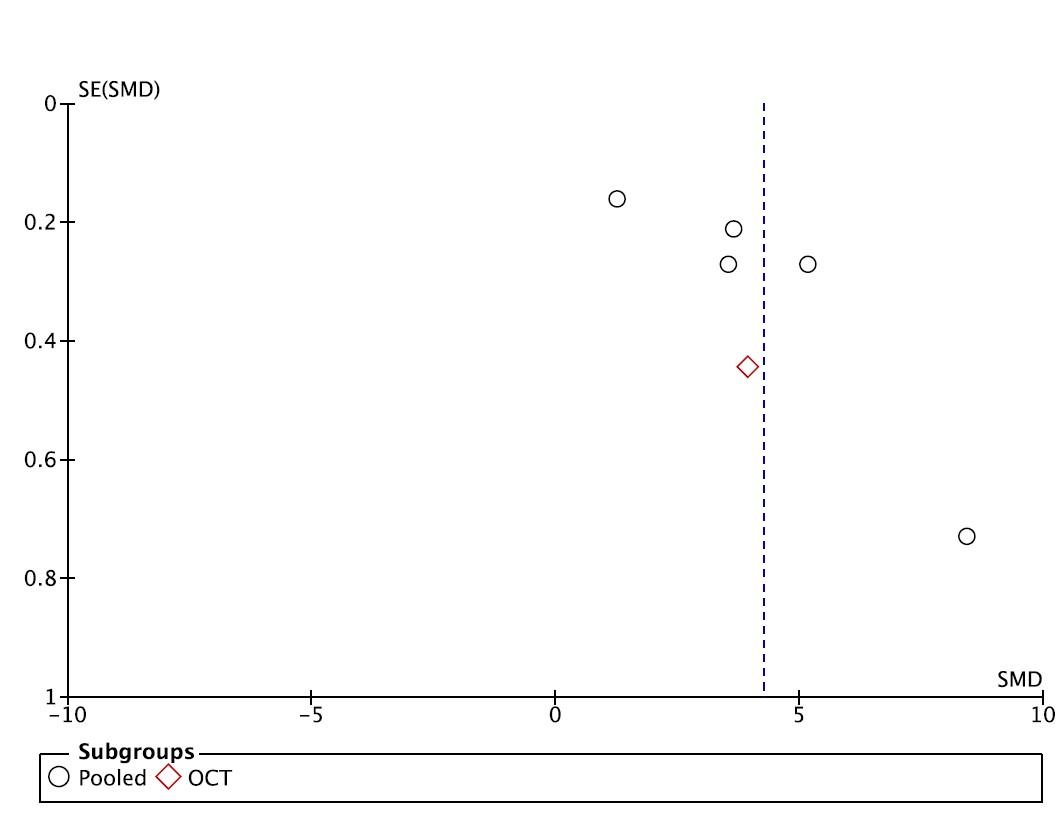

Supplement: Supplementary data 1 [file mmc1.docx]
